# Minor Alterations in Core Promoter Element Positioning Reveal Functional Plasticity of a Bacterial Transcription Factor

**DOI:** 10.1128/mBio.02753-21

**Published:** 2021-11-02

**Authors:** Wamiah P. Chowdhury, Kenneth A. Satyshur, James L. Keck, Patricia J. Kiley

**Affiliations:** a Department of Biomolecular Chemistry, University of Wisconsin—Madison, Madison, Wisconsin, USA; b Integrated Program in Biochemistry, University of Wisconsin—Madison, Madison, Wisconsin, USA; National Cancer Institute

**Keywords:** *Escherichia coli* gene expression, IscR, transcriptional regulation, transcriptional repression

## Abstract

IscR is a global transcription factor that regulates Fe-S cluster homeostasis and other functions in Escherichia coli by either activating or repressing transcription. While the interaction of IscR with its DNA sites has been studied, less is known about the mechanism of IscR regulation of transcription. Here, we show that IscR recruits RNA polymerase to an activated promoter and that IscR binding compensates for the lack of an optimal RNA polymerase σ^70^ −35 promoter element. We also find that the position of the −35 promoter element within the IscR DNA site impacts whether IscR activates or represses transcription. RNA polymerase binding at a distally positioned −35 element within the IscR site results in IscR activation. Molecular modeling suggests that this position of the −35 element allows IscR and RNA polymerase to bind to the promoter from opposite faces of the helix. Shifting the −35 element 1 nucleotide upstream within the IscR binding site results in IscR repression and a steric clash of IscR and RNA polymerase binding in the models. We propose that the sequence similarity of the IscR binding site with the −35 element is an important feature in allowing plasticity in the mechanism of IscR regulation.

## INTRODUCTION

Transcriptional regulation, which is orchestrated by transcription factors and RNA polymerase, is a vital process in all living organisms in order to achieve appropriate levels of gene expression. In bacteria, the core RNA polymerase is composed of β, β′, ω, and two α subunits, and transcription initiation requires core RNA polymerase to associate with a σ specificity factor (1–5). In Escherichia coli, the housekeeping sigma factor, σ^70^, recognizes promoters containing two conserved hexamers that are positioned around bp −10 (^−12^TATAAT^−7^) and bp −35 (^−35^TTGACA^−30^) relative to the transcription start site and are separated by an optimal spacing of 17 bp (6, 7). Regions 2 and 4 of σ^70^ recognize the −10 and −35 promoter elements, respectively, allowing RNA polymerase to bind and initiate transcription (8–10). Some promoters also contain an A/T-rich UP element, which extends from bp −38 to −59 relative to the transcription start site and is recognized by the C-terminal domain (CTD) of the α-subunit of RNA polymerase (11–13). Transcription activators with binding sites overlapping the promoter elements often enhance binding or open complex formation by RNA polymerase when the promoter elements deviate from the consensus sequence (14–17).

The iron-sulfur cluster regulator (IscR) is a conserved global transcription factor that regulates essential functions for Fe-S cluster biogenesis in many bacteria. Under anaerobic conditions, when the demand for Fe-S cluster biogenesis is low, IscR ligates a [2Fe-2S] cluster (holo-IscR) and represses the *isc* operon, which encodes the primary pathway responsible for Fe-S cluster biogenesis as well as IscR (18, 19). Under aerobic conditions, the cluster occupancy of IscR is decreased, derepressing the *isc* operon and further increasing the levels of the cluster-free form of IscR (apo-IscR), which activates the *suf* operon, encoding the alternate Fe-S cluster biogenesis pathway (20–23). This coordinated upregulation of the *suf* and *isc* operons in the presence of O_2_ increases the capacity of Fe-S biogenesis pathways to repair or replace O_2_ or reactive oxygen species (ROS)-damaged Fe-S clusters and maintain Fe-S cluster homeostasis.

In addition to the *isc* and *suf* operons, IscR regulates 40 other genes in E. coli (22). Promoters of these genes contain either a type I or a type II IscR binding site (22). Type I sites are bound only by holo-IscR; thus, promoters containing type I sites (e.g., the *isc* operon) are regulated by IscR primarily under anaerobic conditions (22). Type II sites (e.g., the *suf* operon) can be bound by either apo- or holo-IscR, but promoters containing these sites are regulated primarily under aerobic conditions, in part due to the higher levels of apo-IscR present under these conditions (21, 22). AT-rich tracts flank the site-specific half-sites that distinguish IscR type I from type II sites (22). Type I sites are partially asymmetric, containing 5′-TTGAC on one half-site and 5′-CCGAC on the complementary strand of the other half-site, whereas type II sites are symmetrical, containing 5′-CCxYA (with Y being a pyrimidine) for both half-sites (22). The X-ray crystal structure of the apo-IscR dimer bound to a type II site from the hydrogenase I (*hyaA*) promoter showed that the DNA recognition helix of the winged helix-turn-helix motif inserts into a major groove making base-specific contacts with the IscR half-site and that the wing interacts with the adjacent AT-rich minor groove (24). E43 of the recognition helix plays an important role in discriminating between type I and II sites since the negative charge favors electrostatic interactions with the exocyclic amines of the CC dinucleotides in type II sites but would disrupt binding to type I sites, which contain TT at the corresponding positions in one half-site (24).

All promoters containing known type I sites are repressed by IscR, but promoters containing a type II site can be either repressed or activated. Many IscR type II binding sites are close to or overlapping the −35 hexamer of these σ^70^-dependent promoters. In fact, the position of the IscR binding site that represses the *hyaA* promoter is shifted by only a few nucleotides within the promoter region compared to that of either the *suf* operon or *ydiU*, which are activated by IscR (21, 22, 25). The IscR right half-site has the −35 element embedded within it for all three promoters, with slight variations in the specific spacing of the IscR site relative to the promoter −10 and −35 elements. Thus, subtle differences in promoter architecture must be responsible for directing whether IscR activates or represses promoters.

To understand how promoter architecture affects IscR-dependent transcription regulation, we made mutations in the promoter region that drives the transcription of *ydiU*, a gene encoding a pseudokinase domain that is conserved across all three domains of life (26). While the role of YdiU remains unclear in E. coli, it has been reported recently in Salmonella enterica serovar Typhimurium that the pseudokinase domain plays a role in stress responses through posttranslational protein modification (27, 28). This IscR-activated promoter was chosen because transcriptional activation could be readily quantified using an *in vitro* assay, facilitating analysis. Our approach was to change the position of the −35/−10 promoter elements relative to the IscR binding site, guided by other IscR-regulated promoters, and use *in vitro* transcription assays to test their effect on IscR-dependent transcriptional regulation and basal promoter activity. We also used DNase I footprinting to probe how IscR binding impacts the recruitment of RNA polymerase to activate the wild-type (WT) and mutant promoters. These experiments show that small differences in the position of the −35 promoter element within the IscR binding site play an important role in RNA polymerase recruitment by IscR as well as in determining positive or negative regulation of the promoter by IscR.

## RESULTS

### IscR enhances RNA polymerase binding to the *ydiU* promoter.

**FIG 1:**
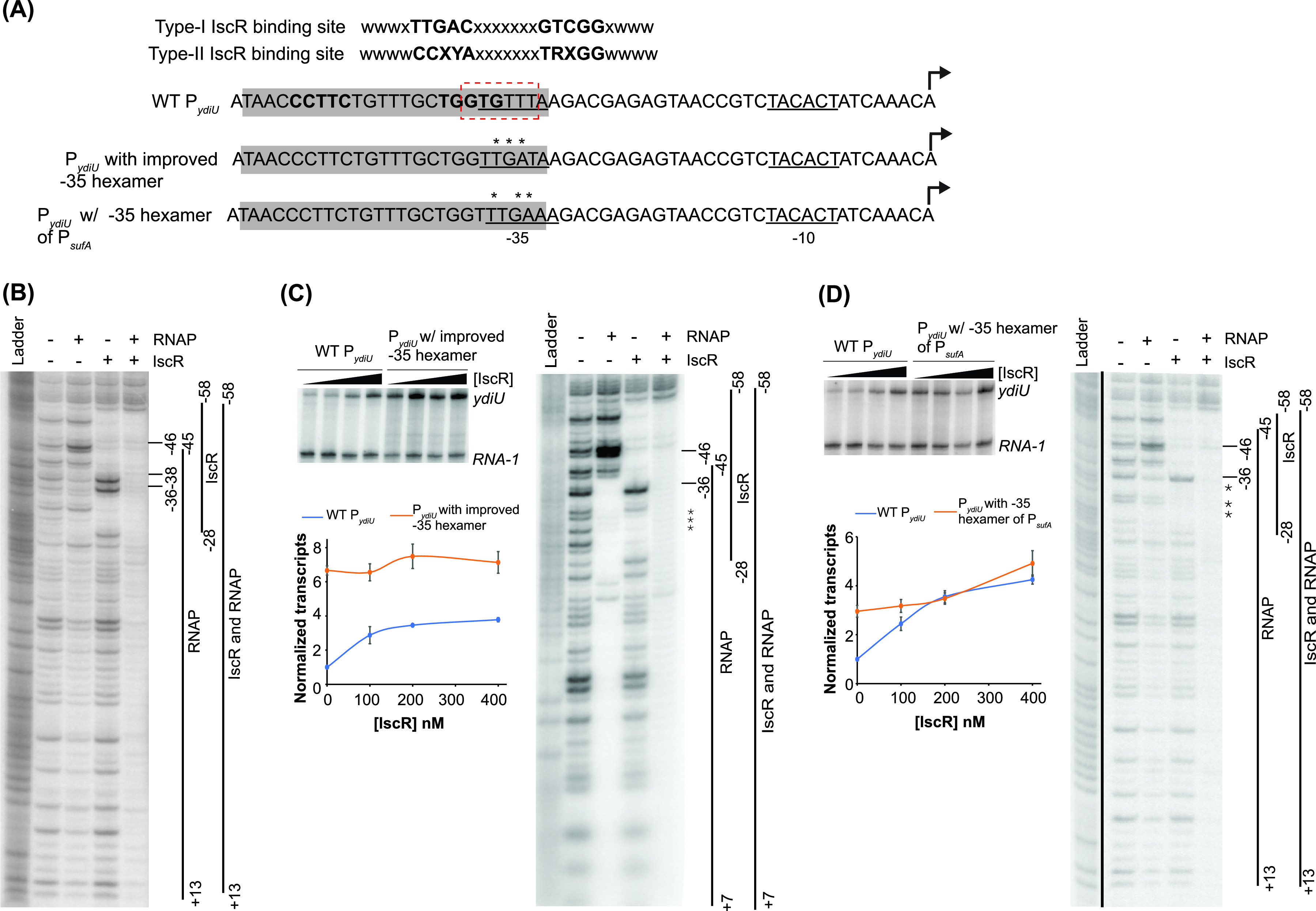
Role of the −35 hexamer in IscR enhancement of RNA polymerase binding and activation of promoters. (A) Consensus type I and II IscR binding sites. The half-sites are highlighted in boldface type. The type II binding sites from IscR-activated WT P*_ydiU_* and its mutants are highlighted in gray. The σ^70^ −35 and −10 promoter elements are underlined, with an alternative −35 hexamer indicated by a red box. The transcription start site is shown with a bent arrow. The engineered mutations in the P*_ydiU_* mutants are marked by asterisks. (B) DNase I footprinting of the P*_ydiU_* fragment from bp −200 to +40 relative to the transcription start site. The regions protected by Eσ^70^ RNA polymerase (RNAP) and IscR are indicated by vertical lines. The hypersensitive sites are marked with bars. The downstream boundary of the RNA polymerase footprint was not defined in these experiments. The ^32^P-radiolabeled top strand of the DNA fragment was incubated with 100 nM Eσ^70^ RNA polymerase, 500 nM IscR, or both, before being subjected to DNase I cleavage and separated by electrophoresis. The Maxam-Gilbert (G+A) ladder is shown in the first lane. (C and D) Comparison of P*_ydiU_* mutants with an altered −35 hexamer. (Left) Representative images following electrophoretic separation of *in vitro* transcription products from plasmid templates containing the region from bp −200 to +40 relative to the transcription start site of WT P*_ydiU_*, P*_ydiU_* with the improved −35 hexamer (C), and P*_ydiU_* with the −35 hexamer of P*_sufA_* (D) in the presence of 50 nM RNA polymerase and 0, 100, 200, or 400 nM IscR. The *ydiU* and control *RNA-1* transcripts are indicated. The transcript levels were quantified by normalizing the *ydiU* transcripts against the control *RNA-1* transcripts. The standard errors from three replicates are shown by the error bars. The lines connecting the data points are not fit to any equation. (Right) DNase I footprinting of the P*_ydiU_* mutants was performed as described above for panel B. The asterisks represent the base pair changes in the mutant promoters.

The recruitment of RNA polymerase to promoters with suboptimal −35 promoter elements is a common mechanism for prokaryotic transcription activation (29, 30). In P*_ydiU_*, 4 of the 6 nucleotides of the −10 hexamer recognized by σ^70^ are conserved (**TA**C**A**C**T** [matches to the consensus are in boldface type]), but two matches to two possible suboptimal −35 hexamers, G**TG**TTT and **T**GTTT**A**, were identified (Fig. 1A). Given the optimal spacing of 17 bp between the −35 hexamer and the −10 hexamer, we considered TGTTTA as the −35 promoter element. Since the −35 hexamer is suboptimal in this promoter, we predicted that IscR might activate transcription of P*_ydiU_* by recruiting RNA polymerase. To test this notion, we carried out a DNase I footprinting assay of the native P*_ydiU_* in the presence of IscR and RNA polymerase.

As expected from previous data (22), IscR protected the region between position −58 and −28 relative to the transcription start site, with hypersensitive sites at −36G and −38T (Fig. 1B). RNA polymerase alone weakly protected the *ydiU* promoter from beyond bp −46 to at least +13 relative to the transcription start site, with a hypersensitive site at position −46. This hypersensitive site is indicative of the αCTD of RNA polymerase interacting with the UP element (12). When both IscR and RNA polymerase were present, the region between −58 and +13 was strongly protected, and the hypersensitive sites were diminished, suggesting that IscR promotes the binding of RNA polymerase to the promoter.

**FIG 2:**
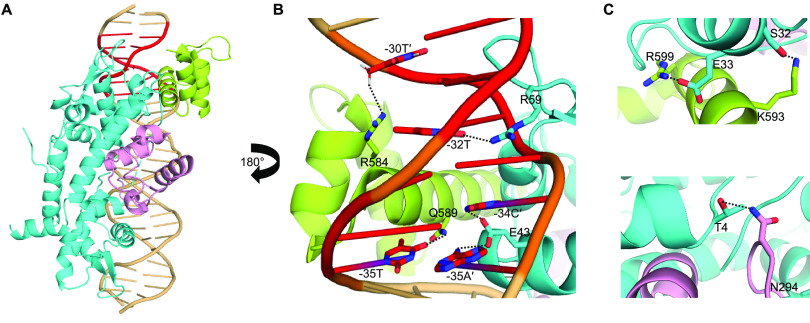
IscR and RNA polymerase likely bind to opposite faces of the *ydiU* promoter. (A) Molecular modeling of IscR and RNA polymerase at the *ydiU* promoter showing the transcription factor and a portion of RNA polymerase binding to opposite faces of the DNA, providing insights into how IscR promotes RNA polymerase recruitment. The IscR dimers are shown in cyan, σ^70^ region 4.2 is shown in green, and the αCTD is shown in pink. The −35 promoter element is highlighted in red. (B) σ^70^ and IscR simultaneously interact with bases of the −35 promoter element. The DNA recognition helix of IscR (cyan) and the −35 promoter element recognition helix of σ^70^ (green) insert into the major groove of the −35 hexamer from opposite faces of the DNA. Q589 and R584 of σ^70^ are positioned to make base-specific interactions with −35T and −30T′ of the −35 promoter element, respectively. E43 of IscR is positioned to interact with the exocyclic amines of −35A′ and −34C′, which are part of the −35 hexamer element, as well as the IscR half-site. R59 of the IscR wing interacts with −32T. (C) Predicted protein-protein interaction between IscR and RNA polymerase. E33 of helix 2 of IscR is in the proximity of R599 of σ^70^, suggesting potential salt bridge formation between the two proteins. S32 of IscR may also H bond with K593 of σ^70^. T4 of IscR may be involved in H bonding with N294 of the αCTD.

To better understand IscR-mediated RNA polymerase recruitment, we modeled the interaction of a portion of RNA polymerase and IscR at the IscR site of the *ydiU* promoter (Fig. 2). We used the X-ray crystal structure of IscR bound to its site at the *hyaA* promoter as a guide to build the model for IscR bound to its site at P*_ydiU_* (24). We reasoned that the IscR interactions at the half-sites of the binding sites from the two promoters would be retained and built the model accordingly. We chose the structure of RNA polymerase bound to the *rpsT* P2 promoter since this promoter has a perfect −35 hexamer as well as a UP element (31). Thus, by superimposing the bases conserved at the −35 hexamer of P*_ydiU_* with those of the *rpsT* P2 promoter, we were able to predict the interaction of RNA polymerase at the *ydiU* promoter. Since the IscR site overlaps the UP and −35 promoter elements, our molecular model focused on the position of IscR relative to the αCTD and σ^70^ region 4.2 of RNA polymerase.

For σ^70^ region 4.2 and IscR, the model suggested that the proteins would bind to opposite faces of the DNA but would be close enough for protein-protein interactions. At the −35 promoter element, which partially overlaps the right half-site of the IscR binding site, the DNA recognition helix of the downstream IscR monomer inserts into the major groove from one face, with the IscR wing inserting into the adjacent minor groove (Fig. 2B). E43 of IscR is positioned to interact with the exocyclic amines of −35A′ and −34C′, similar to the crystal structure of IscR bound to the *hyaA* site (24). R59 of the IscR wing interacts with the AT-rich sequences of the IscR binding site, and a similar interaction could be modeled at the *ydiU* promoter, where the 3′-end AT tract overlaps the −35 promoter element (Fig. 2B). The −35 recognition helix of region 4.2 of σ^70^ is positioned to interact with the −35 promoter element from the face opposite IscR binding, with the two conserved base pairs of the −35 promoter element of P*_ydiU_*, −35T and −30T′, interacting with Q589 and R584 of σ^70^, respectively, as seen at a canonical −35 hexamer (Fig. 2B) (8). Thus, it is likely that IscR and σ^70^ corecognize the −35 hexamer from opposite faces and that the side chains are close enough to interact. Supporting this notion, the model further suggests that there would be an electrostatic interaction between E33 of IscR and R599 of σ^70^, in addition to possible hydrogen bonding between S32 of IscR and K593 (Fig. 2C). One of the αCTD subunits of RNA polymerase is positioned close to the cluster binding domain of the downstream IscR monomer, with possible hydrogen bonding between T4 of the downstream IscR monomer and N294 of the αCTD (Fig. 2C). Thus, this model suggests that a favorable alignment of σ^70^ and IscR bound to the promoter and protein-protein interactions allow improved binding of polymerase to P*_ydiU_* in the presence of IscR.

### An optimal −35 hexamer eliminates the requirement for IscR-dependent activation of P*_ydiU_*.

If IscR activates the transcription of the *ydiU* gene by recruiting RNA polymerase to overcome a weak −35 promoter element, then improving the −35 hexamer should bypass the need for IscR to activate P*_ydiU_*. To test this hypothesis, we replaced the native −35 hexamer with a near-consensus −35 sequence, choosing nucleotides that would not disrupt IscR binding (Fig. 1A). Transcription activation was measured using an *in vitro* transcription assay with isolated RNA polymerase, IscR, and a plasmid DNA template bearing the relevant promoter sequence (Fig. 1C). Transcript levels produced by P*_ydiU_* or its mutant were normalized to that of a plasmid-generated RNA-1 transcript that is not regulated by IscR (22). For the wild-type promoter, IscR increased the amount of the *ydiU* transcript in an IscR concentration-dependent manner, resulting in 4-fold transcription activation at the highest IscR concentration tested (Fig. 1C; see also Fig. S1A in the supplemental material). Improving the −35 hexamer significantly increased the basal activity of this promoter to a level almost 7-fold higher than that of wild-type P*_ydiU_*. Transcription was only slightly increased at the highest level of IscR tested, suggesting that the increased promoter strength produced by the improved −35 element bypassed the IscR-dependent step in transcription activation of the native promoter.

10.1128/mBio.02753-21.1 FIG S1 Replicates of *in vitro* transcription assays of P*_ydiU_* with the improved −35 hexamer (A) and with the −35 hexamer of P*_sufA_* (B). Shown are gel images following electrophoretic separation of *in vitro* transcription products from plasmids containing the region from bp −200 to +40 relative to the transcription start site of wild-type P*_ydiU_* and P*_ydiU_* variants in the presence of 50 nM RNA polymerase and 0, 100, 200, or 400 nM IscR. The *ydiU* and control RNA-1 transcripts are indicated. Download FIG S1, PDF file, 0.9 MB. Copyright © 2021 Chowdhury et al. 2021 Chowdhury et al. https://creativecommons.org/licenses/by/4.0/ This content is distributed under the terms of the Creative Commons Attribution 4.0 International license.

RNA polymerase binding to the improved promoter was assayed by DNase I footprinting (Fig. 1C). Consistent with an improved −35 hexamer, the RNA polymerase binding affinity to the promoter was enhanced since maximal protection of the promoter region was now observed at RNA polymerase concentrations that only weakly protected the native promoter. The enhanced binding of RNA polymerase to the improved promoter was similar to that of the native promoter when IscR was also present, likely explaining why IscR had no additional effect in the *in vitro* transcription assays for the improved promoter. The IscR protection pattern of the improved promoter was nearly identical to that observed for the native promoter, except for changes to the hypersensitive sites at position −36 and −38, indicating minor alterations of IscR interaction with its site. Nevertheless, when both proteins were present, protection by both IscR and RNA polymerase was observed, indicating that both proteins can bind simultaneously to the promoter. Altogether, the *in vitro* transcription and footprinting assays show that IscR compensates for a suboptimal −35 hexamer in activating P*_ydiU_* and that IscR is no longer required when the suboptimal −35 hexamer is replaced with a near-consensus sequence.

### IscR can also recruit RNA polymerase when the −35 hexamer of P*_ydiU_* is replaced with that of P*_sufA_*.

To determine if compensation for a suboptimal −35 element by IscR is common to other IscR-activated promoters, we exchanged just the −35 promoter element of P*_ydiU_* with that of P*_sufA_* (Fig. 1A). The −35 hexamer of the native P*_sufA_* is shifted 1 nucleotide closer to the −10 element than the configuration of P*_ydiU_*, whereas the spacing of the IscR binding site relative to the −10 element is the same for both promoters. The sequence of the P*_sufA_* −35 hexamer is also more conserved than that of P*_ydiU_*, with 4 nucleotides, **TTGA**AC (highlighted in boldface type), matching the consensus hexamer. The basal level of transcript produced from the P*_ydiU_* mutant with the P*_sufA_* −35 hexamer positioned 1 nucleotide closer to the −10 element was 3-fold higher than that of the native *ydiU* promoter, presumably a result of the stronger −35 hexamer. IscR was also able to activate the promoter albeit to a lesser extent than the native P*_ydiU_* promoter (Fig. 1D and Fig. S1B).

**FIG 3:**
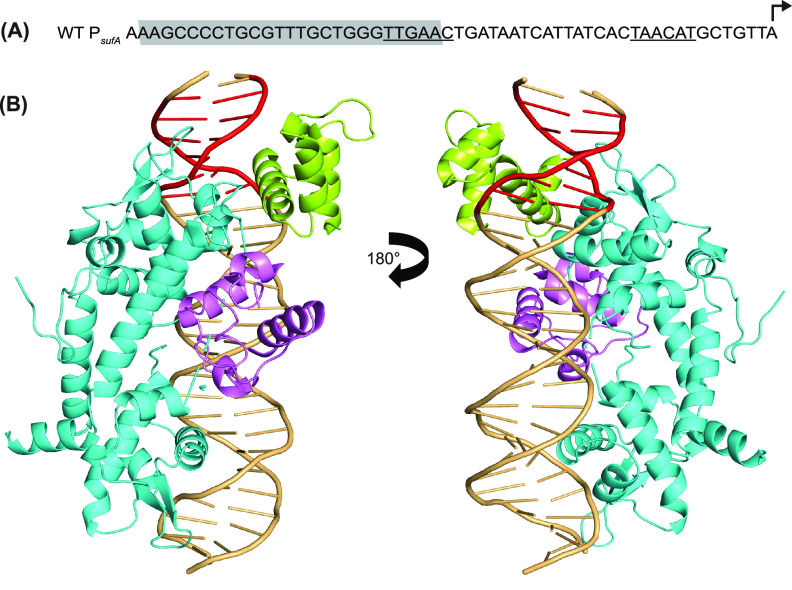
The IscR-RNA polymerase interaction at the *sufA* promoter is likely similar to that observed at the *ydiU* promoter. (A) Sequence of the IscR-activated *sufA* promoter, with the type II IscR binding site highlighted in gray, the σ^70^ −35 and −10 promoter elements underlined, and the transcription start site shown with a bent arrow. (B) Molecular modeling of IscR and RNA polymerase at the *sufA* promoter showing the transcription factor and a portion of RNA polymerase binding to opposite faces of the DNA, similar to the model at the *ydiU* promoter. The IscR dimers are shown in cyan, σ^70^ region 4.2 is shown in green, and the αCTD is shown in pink. The −35 promoter element is highlighted in red.

DNase I footprinting of the *sufA* −35 mutant of P*_ydiU_* showed that RNA polymerase protection was increased compared to that of the native promoter but not as strongly as that of the near-consensus −35 element (Fig. 1D). RNA polymerase protection was enhanced upon the addition of IscR, indicating that IscR could still recruit RNA polymerase, even with the shorter spacing between the −35 and −10 promoter elements and a 1-nucleotide shift of the −35 promoter element downstream relative to the IscR binding site. Molecular modeling of IscR and RNA polymerase at the IscR binding site of WT P*_sufA_* indicates that the two proteins bind to opposite faces of the DNA for activation, similar to the model of IscR-RNA polymerase binding at P*_ydiU_* (Fig. 3). Therefore, the −35 hexamer of P*_sufA_* is also in a position favorable to promote interactions between IscR and RNA polymerase for transcription activation.

### Increasing the spacing between the IscR binding site and the promoter elements decreases the transcription regulation of *ydiU*.

**FIG 4:**
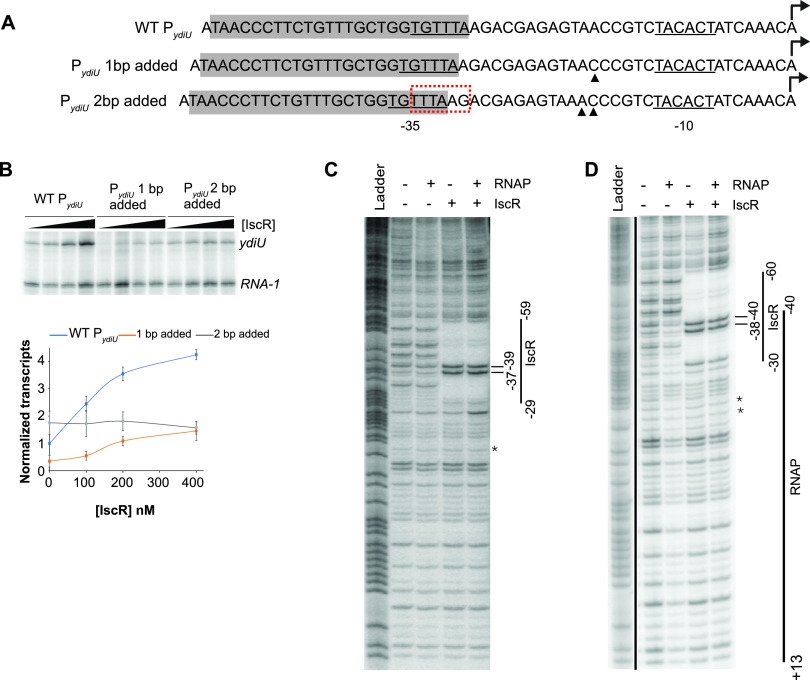
The position of the IscR binding site relative to the promoter elements affects *ydiU* regulation. (A) Sequence comparison of WT P*_ydiU_* with mutant promoters containing a 1-bp or 2-bp addition. The IscR binding sites are highlighted in gray. The addition of bases is indicated by the filled triangles. The σ^70^ promoter elements are underlined, and the transcription start sites are shown with arrows. The alternative −35 hexamer is marked by the red box. (B) Representative image following electrophoretic separation of *in vitro* transcription products from plasmid templates containing the region from bp −200 to +40 relative to the transcription start site of WT and mutant P*_ydiU_* with 1 and 2 bp added, in the presence of 50 nM RNA polymerase and 0, 100, 200, or 400 nM IscR. The *ydiU* and control *RNA-1* transcripts are indicated. The *ydiU* transcript levels are normalized against the control *RNA-1* transcripts. The standard errors from the three replicates are shown by the error bars. The lines connecting the data points are not fit to any equation. (C and D) DNase I footprinting of P*_ydiU_* mutants containing a 1-bp addition (C) and a 2-bp addition (D) and the region between bp −200 and +40 relative to the transcription start site. The region protected by IscR is indicated by the vertical line. The hypersensitive sites are marked with bars. The asterisk represents the location of the added base pair. The downstream boundary of the RNA polymerase footprint was not defined in these experiments. The ^32^P-radiolabeled top strands of the DNA fragments were incubated with 100 nM RNA polymerase, 500 nM IscR, or both, before being subjected to DNase I cleavage and separated by electrophoresis. The Maxam-Gilbert (G+A) ladders are shown in the first lanes.

To test how flexible the spacing requirements are between IscR and the promoter elements, we increased the spacing between the IscR binding site/−35 hexamer and the −10 element of P*_ydiU_* by 1 or 2 bp (Fig. 4A). Since the −35 hexamer is embedded within the IscR binding site, the spacing between these two sequences was unchanged in the spacing mutants. The addition of 1 bp greatly reduced the basal level of transcription from the promoter as evidenced by the very low transcript level with RNA polymerase alone (Fig. 4B and Fig. S2). Adding IscR to the assay mix increased transcription only weakly, with 3.5-fold-lower transcript levels than those of the native promoter at all concentrations of IscR tested (Fig. 4B and Fig. S2). Since the −35 element was unchanged, the decrease in transcription activation likely results from the lengthening of the spacing between the −35 and −10 elements, which deviates from the optimal 17-bp spacing. The addition of 2 bp in the spacer region slightly increased the basal level of transcription by 1.5-fold, suggesting the usage of an alternative −35 promoter element (Fig. 4B and Fig. S2). The new −35 hexamer, **TT**T**A**AG, has 3 matches to the consensus −35 hexamer, compared to the two present in the native promoter, **T**GTTT**A** (Fig. 4A). Adding IscR had no effect on transcript levels, suggesting that the altered position of the −35 hexamer relative to the IscR binding site no longer allowed the recruitment of RNA polymerase by the transcription factor (Fig. 4B and Fig. S2).

10.1128/mBio.02753-21.2 FIG S2 Replicates of *in vitro* transcription assays of P*_ydiU_* mutants with 1 and 2 bp added. Shown are gel images following electrophoretic separation of *in vitro* transcription products from plasmids containing the region from bp −200 to bp +40 relative to the transcription start site of the WT and P*_ydiU_* mutants containing additional base pairs, in the presence of 50 nM RNA polymerase and 0, 100, 200, or 400 nM IscR. The *ydiU* and the control RNA-1 transcripts are indicated. Download FIG S2, PDF file, 0.8 MB. Copyright © 2021 Chowdhury et al. 2021 Chowdhury et al. https://creativecommons.org/licenses/by/4.0/ This content is distributed under the terms of the Creative Commons Attribution 4.0 International license.

DNase I footprinting was used to determine whether these mutations also affected RNA polymerase recruitment (Fig. 4C and D). Consistent with the low levels of transcripts observed in the transcription assay, no or little protection by RNA polymerase was observed in either the presence or absence of IscR when the IscR binding site was moved 1 or 2 bp away from the −10 promoter element. Taken together, these data suggest that increasing the distance between the −10 element and the −35 element embedded within the IscR site, as well as a 2-bp shift in the position of the −35 hexamer within the IscR binding site of P*_ydiU_*, results in a loss of recruitment of RNA polymerase by IscR and a loss of the IscR-dependent transcription activation of *ydiU*.

### Decreased spacing between the IscR binding site and the −10 promoter element converts an IscR-activated promoter to a repressed promoter.

**FIG 5:**
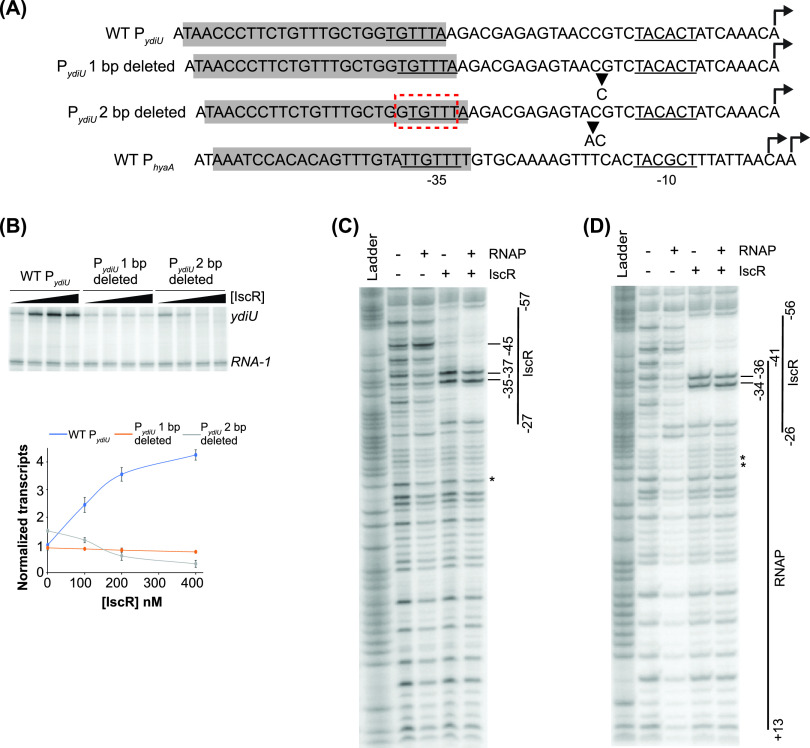
Decreased spacing between the IscR binding site and the −10 promoter element converts an IscR-activated promoter to a repressed promoter. (A) Sequence comparison of WT P*_ydiU_* with mutants containing 1-bp and 2-bp deletions. The IscR binding sites are highlighted in gray. The base pair deletions are indicated by the inverted triangles. The σ^70^ promoter elements are underlined, and the transcription start sites are shown with arrows. Alternative −35 promoter elements are marked in red boxes. (B) Representative image following electrophoretic separation of *in vitro* transcription products from plasmid templates containing the region from bp −200 to +40 relative to the transcription start site of WT and mutant P*_ydiU_* with 1- and 2-bp deletions, in the presence of 50 nM RNA polymerase and 0, 100, 200, or 400 nM IscR. The *ydiU* and control *RNA-1* transcripts are indicated. The *ydiU* transcript levels are normalized against the control *RNA-1* transcript level. Standard errors from three replicates are shown by the error bars. The lines connecting the data points are not fit to any equation. (C and D) DNase I footprinting of mutant P*_ydiU_* containing 1-bp (C) and 2-bp (D) deletions and the region between bp −200 and +40 relative to the transcription start site. The regions protected by RNA polymerase and IscR are indicated by the vertical lines. The hypersensitive sites are marked with bars. The asterisk represents the location of the deleted base pair. The downstream boundary of the RNA polymerase footprint was not defined in these experiments. The ^32^P-radiolabeled top strands of the DNA fragments were incubated with 100 nM RNA polymerase, 500 nM IscR, or both, before being subjected to DNase I cleavage and separated by electrophoresis. The Maxam-Gilbert (G+A) ladders are shown in the first lanes.

The distance between the IscR binding site and the −10 element in the IscR-repressed *hyaA* promoter is shorter than that in P*_ydiU_* by 2 bp. The −35 element of P*_hyaA_* is also embedded within the IscR site but is shifted 1 nucleotide upstream relative to P*_ydiU_*. To examine the flexibility of the position of the IscR binding site relative to the promoter elements in activation versus repression, we made 1- or 2-bp deletions in the spacer region of P*_ydiU_* between the −35 and −10 promoter elements (Fig. 5A). Compared to the native *ydiU* promoter, when the IscR binding site was moved 1 nucleotide toward the −10 element, there was a loss of IscR-dependent regulation in the *in vitro* transcription assay (Fig. 5B and Fig. S3). DNase I footprinting of the mutant promoter (Fig. 5C) showed little protection by RNA polymerase. When IscR was added, only protection by IscR was observed, indicating that IscR no longer promoted RNA polymerase binding.

10.1128/mBio.02753-21.3 FIG S3 (A) Sequence comparison of the WT and P*_ydiU_* mutants with a 2-bp deletion between the IscR binding site and the −10 promoter element. The IscR binding site is highlighted in gray. The σ^70^ −35 and −10 promoter elements are underlined, with an alternative −35 hexamer indicated by a red box. The transcription start site is shown with a bent arrow. The inverted triangles show the deleted base pairs. (B) Representative image following electrophoretic separation of *in vitro* transcription products from plasmid templates containing the region from bp −200 to +40 relative to the transcription start site of WT P*_ydiU_* and P*_ydiU_* mutants with 2-bp deletions in the presence of 50 nM RNA polymerase and 0, 100, 200, or 400 nM IscR. The *ydiU* and control RNA-1 transcripts are indicated. (C) Replicates of *in vitro* transcription assays of P*_ydiU_* mutants with 1- or 2-bp deletions. The assay was performed as described above for panel B. Download FIG S3, PDF file, 1.2 MB. Copyright © 2021 Chowdhury et al. 2021 Chowdhury et al. https://creativecommons.org/licenses/by/4.0/ This content is distributed under the terms of the Creative Commons Attribution 4.0 International license.

However, when the binding site was moved 2 nucleotides closer to the −10 element, a position similar to that of the repressed P*_hyaA_* promoter, the mutant promoter was converted from one that was activated by IscR to one that was repressed, with a 5-fold decrease in the transcript level at the highest level of IscR tested (Fig. 5B and Fig. S3C). This switch to an IscR-repressed promoter was not dependent on which sequences were deleted since the removal of two different pairs of nucleotides yielded the same result (Fig. S3A and B). The IscR site of P*_ydiU_* contains overlapping weak −35 elements, suggesting that the −35 hexamer used by RNA polymerase in these mutant constructs could also be affected by the deletion of the nucleotides. Indeed, the deletion of 2 nucleotides results in a small but reproducible 1.5-fold increase in the basal level of transcription. The latter result is consistent with using 5′-G**TG**TTT (matches two [highlighted in boldface type] of the three most conserved nucleotides in the −35 hexamer), which is shifted 1 nucleotide upstream compared to the native −35 hexamer, 5′-**T**GTTT**A**, of the native promoter. The length of the RNA polymerase-protected region by DNase I footprinting (Fig. 5D) was the same as that in the native promoter. With both IscR and RNA polymerase, only protection by IscR was observed, indicating that IscR binding occludes RNA polymerase binding. Taken together, these results suggest that small shifts in the positioning of the promoter elements relative to the IscR binding site determine whether IscR activates or represses the transcription of promoters containing type II IscR binding sites.

### The position of the −35 promoter element within the IscR binding site determines IscR-dependent regulation.

**FIG 6:**
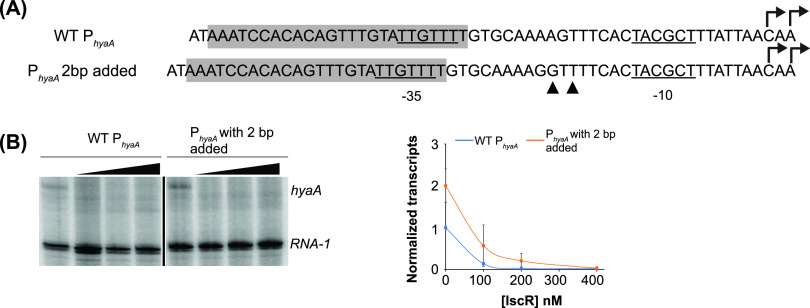
The position of the IscR binding site relative to the −10 promoter element does not influence transcription regulation by IscR. (A) Sequence comparison of WT P*_hyaA_* with the mutant promoter containing a 2-bp addition. The IscR binding sites are highlighted in gray. The addition of bases is indicated by the filled triangles. The σ^70^ promoter elements are underlined, and the transcription start sites are shown with arrows. (B) Representative image following electrophoretic separation of *in vitro* transcription products from plasmid templates containing the region from bp −200 to +40 relative to the transcription start site of WT P*_hyaA_* and mutant P*_hyaA_* with 2 bp added, in the presence of 50 nM RNA polymerase and 0, 100, 200, or 400 nM IscR. The *hyaA* and control *RNA-1* transcripts are indicated. The *hyaA* transcript levels are normalized against the control *RNA-1* transcript level. Standard errors from three replicates are shown by the error bars. The lines connecting the data points are not fit to any equation.

If the position of the −35 element relative to the IscR binding site is driving repression by IscR, then moving the IscR site and the associated −35 element of P*_hyaA_* upstream by 2 bp should maintain IscR repression. To test this, we made a mutant of P*_hyaA_* where we increased the spacing between the IscR binding site and the −10 hexamer by 2 bp (Fig. 6A). This changed the register of the IscR binding site relative to the −10 hexamer to be the same as that of IscR-activated *P_ydiU_* while maintaining the same register of the binding site relative to the −35 hexamer as that of the native *hyaA* promoter. An *in vitro* transcription assay showed that the mutant is repressed by IscR, similar to WT P*_hyaA_*, indicating that small changes in the spacing between IscR and the −10 hexamer do not influence transcription regulation and that it is the positioning of the −35 hexamer within the IscR binding site that dictates whether promoters are positively or negatively regulated by IscR (Fig. 6B and Fig. S4).

10.1128/mBio.02753-21.4 FIG S4 Replicates of *in vitro* transcription assays of WT P*_hyaA_* and the P*_hyaA_* mutant with 2 bp added. Shown are gel images following electrophoretic separation of *in vitro* transcription products from plasmids containing the region from bp −200 to bp +40 relative to the transcription start site of the WT and the P*_hyaA_* mutant in the presence of 50 nM RNA polymerase and 0, 100, 200, or 400 nM IscR. The *hyaA* and the control RNA-1 transcripts are indicated. Download FIG S4, PDF file, 1.3 MB. Copyright © 2021 Chowdhury et al. 2021 Chowdhury et al. https://creativecommons.org/licenses/by/4.0/ This content is distributed under the terms of the Creative Commons Attribution 4.0 International license.

**FIG 7:**
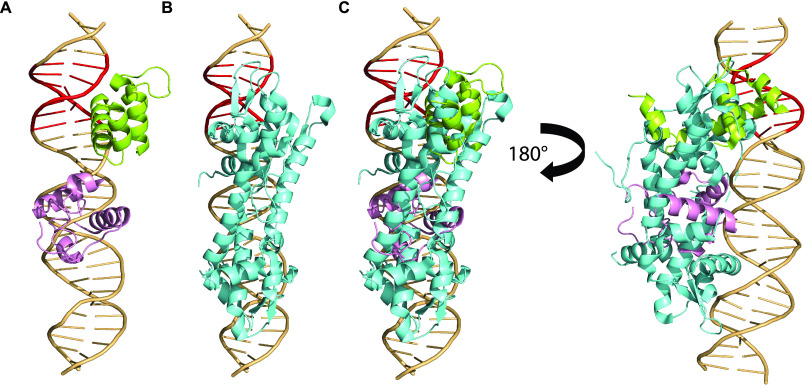
The orientation of IscR relative to RNA polymerase is determined by the location of the −35 promoter element embedded within the IscR binding site. (A) Molecular modeling of σ^70^ and αCTD binding at the IscR binding site/promoter elements of P*_hyaA_*, with σ^70^ region 4.2 shown in green and the αCTD shown in pink. The −35 promoter elements are highlighted in red. (B) IscR, shown in cyan, binds to the promoter at the UP and the −35 promoter element. (C) Modeling of both IscR and RNA polymerase at the promoter showing that the two proteins occupy the same space within the promoter, suggesting extensive steric clashes.

Since our modeling of IscR and RNA polymerase at the IscR binding site of the *ydiU* promoter suggested that the positioning of the −35 promoter element within the IscR binding site allowed favorable binding of both IscR and the σ^70^ subunit of RNA polymerase, we investigated whether the change in the positioning of the −35 element in P*_hyaA_* predicted unfavorable interactions. We built a similar molecular model of IscR and RNA polymerase interactions but at the IscR-repressed *hyaA* promoter (Fig. 7). The model shows extensive clashes between the transcription factor and the σ^70^ subunit of RNA polymerase, in contrast to the compatible orientation predicted at the *ydiU* promoter. The DNA recognition helix of the downstream IscR monomer occupied the same space on the DNA as the −35 hexamer recognition helix of σ^70^. Clashes were also evident between other helices of the IscR monomer and region 4.2 of σ^70^, suggesting that IscR and σ^70^ cannot simultaneously occupy the −35 promoter element at the *hyaA* promoter. The upstream IscR monomer showed clashes with the αCTD of RNA polymerase, providing further evidence that RNA polymerase and IscR cannot bind to the *hyaA* promoter at the same time and also explaining the loss of increased RNA polymerase binding in the presence of IscR, observed at the *ydiU* promoter mutant with the altered −35 hexamer location. Thus, subtle differences in the position of the −35 promoter element within the IscR binding site change the position of IscR relative to RNA polymerase, disrupting IscR-enhanced RNA polymerase binding.

## DISCUSSION

The global transcription factor IscR is able to regulate promoters containing either a type I or II binding site. Here, we investigate the mechanism of IscR activation for a promoter containing a type II binding site. Our results show that IscR recruits σ^70^ RNA polymerase to P*_ydiU_*, which contains a suboptimal −35 promoter element. Enhanced RNA polymerase binding by IscR is dependent on the spacing between the IscR binding site and the promoter elements. Furthermore, the relative spacing of the −35 hexamer within the IscR binding site plays an important role in determining whether IscR activates or represses a promoter, providing a flexible platform for evolving IscR-regulated promoters.

The structural prediction afforded by the modeling of RNA polymerase and IscR at P*_ydiU_* and P*_hyaA_* points to the importance of the position of the −35 promoter element within the IscR binding site for appropriate transcriptional regulation. In IscR-repressed P*_hyaA_*, the position of the −35 promoter element likely prevents IscR and RNA polymerase from simultaneously occupying the promoter, resulting in repression by RNA polymerase occlusion. In IscR-activated P*_ydiU_*, the position of the −35 promoter element within the IscR binding site is such that IscR and RNA polymerase can occupy the promoter from opposite faces of the DNA, allowing the proteins to bind simultaneously. The binding of IscR and σ^70^ region 4.2 from opposite faces of the DNA at the −35 promoter element is reminiscent of the interaction between the cI transcription factor of bacteriophage λ and σ^70^ region 4.2 at the P_RM_ promoter, which also contains a suboptimal −35 promoter element embedded within the cI binding site (32). The precise location of the binding site relative to the −35 promoter element allows the appropriate positioning of λcI and σ^70^ for favorable interactions to take place (32–34). Similar interactions between IscR and σ^70^ region 4.2 could explain the requirement of the −35 hexamer position within the IscR binding site of P*_ydiU_* and P*_sufA_* for activation.

**FIG 8:**
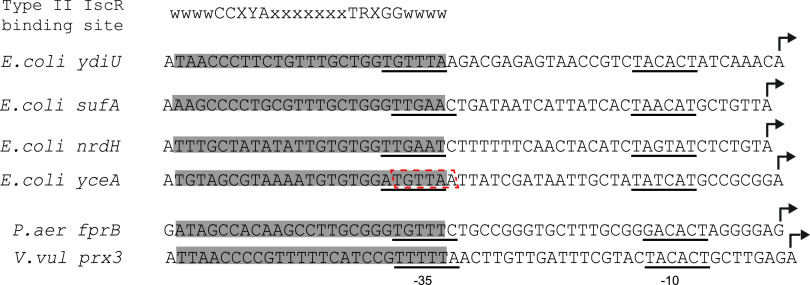
The position of the IscR binding site relative to the −35/−10 promoter elements is conserved in IscR-activated promoters in E. coli and other gammaproteobacteria. The type II IscR binding sites of IscR-activated promoters are highlighted in gray. The −35 and −10 promoter elements are underlined, with the alternative −35 hexamer shown in the red box. The transcription start sites of the promoters are indicated by bent arrows.

The position of the −35 hexamer within the IscR binding site in P*_ydiU_* is conserved in three other IscR-activated promoters in E. coli (Fig. 8). Although the −35 hexamer in the *sufA* promoter is shifted 1 nucleotide downstream within the IscR binding site compared to P*_ydiU_*, we have shown that IscR can promote RNA polymerase binding to activate the promoter from the shifted position of the −35 promoter element. The promoter of the *nrdHIEF* operon, which encodes a ribonucleotide reductase, as well as the promoter of the *yceA* (*trhO*) gene, which is involved in tRNA hydroxylation, are also activated by IscR from a type II IscR binding site, and the position of the −35 promoter element within the binding site of these promoters is the same as that in P*_ydiU_* or P*_sufA_* (Fig. 8) (35). This positioning of the −35 promoter hexamer within the IscR binding site is conserved in other gammaproteobacteria as well. In Vibrio vulnificus, the *prx3* promoter of a peroxiredoxin-encoding gene is activated by IscR from a type II binding site, and the −35 promoter element embedded within the IscR binding site is in the same position as that in P*_sufA_* (Fig. 8) (36). A similar positioning of the −35 hexamer within a type II IscR binding site is also found within the IscR-activated ferredoxin NADP^+^ reductase gene, *fprB*, of Pseudomonas aeruginosa (Fig. 8) (37). The conserved location of the −35 promoter element within the IscR binding site of these activated promoters highlights the importance of this positioning in IscR-dependent activation. It is likely that in all of these activated promoters, IscR and RNA polymerase adopt the same orientation as that in the *ydiU* promoter, indicating that there is a small window of sequence space where the position of the −35 hexamer allows compatible alignment of IscR and RNA polymerase to activate transcription.

Class II transcription activators have binding sites centered around position −41.5 relative to the transcription start site and include members of the CRP/FNR family of transcription factors (16, 38, 39). At this location, transcription factors are able to interact with both the α and σ^70^ subunits of RNA polymerase to activate transcription (34, 40–46). The IscR binding sites in P*_ydiU_* and other IscR-activated promoters in E. coli are located at similar positions, which may allow IscR to have similar interactions with RNA polymerase. Given the overlap between the IscR binding site and the predicted UP element in P*_ydiU_*, and based on our modeling of the two proteins at the *ydiU* promoter, αCTD and IscR may interact with each other. The overlap of the IscR binding site and the −35 promoter element also suggests an interaction between IscR and σ^70^, as has been observed at other class II promoters (34, 40, 41, 46, 47). This prediction provides ground for future work.

Class II activators are also known to compensate for the lack of a recognizable −35 promoter element at the promoters that they regulate (48–51). We have shown that IscR compensates for the lack of an optimal −35 hexamer at the *ydiU* promoter, making it even more likely that IscR acts as a class II activator. Region 4.2 of σ^70^ is known to make base-specific contacts at each of the 6 bp of a canonical −35 hexamer (8). Since *ydiU* contains only the first and last base pairs of a canonical −35 hexamer, σ^70^ interactions at the −35 promoter element are likely not optimal. IscR may compensate for this suboptimality by stabilizing σ^70^ binding at the −35 promoter element. Because the right IscR half-site partially overlaps the −35 promoter element, IscR and σ^70^ are brought into close contact with each other, presumably allowing direct protein-protein interactions to take place. Such interactions likely appropriately position region 4.2 of σ^70^ at the −35 promoter element, stabilize RNA polymerase-DNA interactions, and could increase the rate of subsequent steps in transcription initiation, as has been seen for other class II activators (33, 34, 47).

IscR inefficiently activates and represses promoters containing a type II IscR binding site under anaerobic conditions, compared to aerobic conditions. This difference in regulation may be explained by conformational changes within the transcription factor upon [2Fe-2S] cluster binding, which may change IscR-RNA polymerase interactions at promoters with type II sites. Amino acid side chains of apo-IscR involved in making favorable interactions with RNA polymerase may undergo conformational changes in holo-IscR that disrupt protein-protein interactions, negatively impacting RNA polymerase recruitment by IscR and resulting in defective activation of type II promoters, such as *ydiU* and *sufA*, under anaerobic conditions. However, at the repressed *hyaA* promoter, these conformational changes within holo-IscR may alleviate some of the steric clashes between apo-IscR and RNA polymerase, giving RNA polymerase a chance to bind to the promoter and initiate transcription, likely explaining the poor repression of *hyaA* by IscR under anaerobic conditions. Once E. coli switches from anaerobic to aerobic conditions, apo-IscR levels increase, and the transcription factor is in a conformation that is able to more efficiently regulate the promoters containing type II IscR binding sites. Thus, it seems likely that by evolving a type II IscR binding site overlapping the promoter elements, E. coli is able to link IscR cluster binding to the proper regulation of these promoters through changes in IscR-RNA polymerase interactions. This allows E. coli to elicit the appropriate response to oxygen, based on the cluster occupancy state of IscR.

In summary, our work reveals that minor alterations in the position of the σ^70^ −35 promoter element embedded within the IscR binding site allow functional plasticity of the transcription factor. The sequence similarity between the −35 promoter element and the IscR right half-site allows the partial overlap of the two sites, which brings RNA polymerase and IscR in proximity to each other at these promoters. We propose that a favorable alignment of the −35 hexamer within the IscR right half-site promotes RNA polymerase recruitment by IscR and transcription activation. In contrast, small shifts in the position of the −35 hexamer within the IscR binding site can reorient the transcription factor relative to RNA polymerase, causing a steric clash and repression. Moreover, all characterized promoters to date containing a type II IscR binding site, activated or repressed, have suboptimal promoter elements, consistent with weak interactions between the promoter and RNA polymerase, which can be exploited by IscR for RNA polymerase recruitment in the case of activation or RNA polymerase displacement for repression. Thus, the evolution of the IscR binding site overlapping the promoter elements has allowed the transcription factor to differentially regulate promoters based on subtle differences in spacing between its binding site and the −35 promoter element. This in turn allows E. coli to use a single transcription factor to regulate a number of genes in response to oxygen.

## MATERIALS AND METHODS

### Plasmid construction.

The region between bp −200 and bp +40 relative to the transcription start site of P*_ydiU_* was PCR amplified from the chromosomal DNA of E. coli MG1655, with primers containing XhoI and BamHI sites in the flanking regions. The resulting fragment was cloned into pPK7179 (52) containing the same restriction enzyme sites. The resulting plasmid was used as a template for QuikChange site-directed mutagenesis (Stratagene) to make the P*_ydiU_* promoter mutants (see Table S1 in the supplemental material). Mutants of P*_hyaA_* were created by QuikChange site-directed mutagenesis (Stratagene) using the plasmid pPK6842, which contains the region between bp −200 and bp +40 relative to the transcription start site of P*_hyaA_* (22). Primers used to construct the mutants are listed in Table S2.

10.1128/mBio.02753-21.5 TABLE S1 List of plasmids and E. coli strains used in this study. Download Table S1, PDF file, 0.3 MB. Copyright © 2021 Chowdhury et al. 2021 Chowdhury et al. https://creativecommons.org/licenses/by/4.0/ This content is distributed under the terms of the Creative Commons Attribution 4.0 International license.

10.1128/mBio.02753-21.6 TABLE S2 List of primers used to construct P*_ydiU_* and P*_hyaA_* mutants. The XhoI and BamHI sites in the primers used for cloning WT *ydiU* into pPK7179 are underlined. Download Table S2, PDF file, 0.1 MB. Copyright © 2021 Chowdhury et al. 2021 Chowdhury et al. https://creativecommons.org/licenses/by/4.0/ This content is distributed under the terms of the Creative Commons Attribution 4.0 International license.

### *In vitro* transcription assays.

Plasmids were purified using a QIAfilter maxi kit (Qiagen). A total of 2 nM supercoiled plasmid DNA was incubated with 100, 200, or 400 nM IscR; 0.25 μCi of [α-^32^P]UTP (3,000 μCi/mmol; PerkinElmer); 20 μM UTP; and 500 μM (each) ATP, GTP, and CTP for 20 min at 37°C in a solution containing 40 mM Tris (pH 7.9), 50 mM KCl, 10 mM MgCl_2_, 100 μg/ml bovine serum albumin (BSA), and 0.1 mM dithiothreitol (DTT). E. coli RNA polymerase with σ^70^ (New England BioLabs [NEB]) was added to the assay mixtures at a 50 nM final concentration, and the reactions were terminated after 5 min by adding stop solution (USB). The samples were heated to 90°C for 60 s before being electrophoresed on a 7 M urea–8% polyacrylamide gel in 0.5× Tris-borate-EDTA (TBE) buffer. The transcripts were visualized using a Typhoon FLA-9000 gel imaging scanner and quantified using AzureSpot analysis software (Azure Biosystems).

### DNase I footprinting.

DNA fragments containing P*_ydiU_*, P*_hyaA_*, or their mutant derivatives were isolated from the relevant plasmids following digestion with BamHI and HindIII. The 3′ end of the top strand was radiolabeled with [α-^32^P]dGTP (PerkinElmer) using Klenow fragment. The radiolabeled fragment was isolated from a 5% acrylamide gel, purified using a QIAquick gel extraction kit, and incubated with either 100 nM E. coli RNA polymerase with σ^70^ (NEB), 500 nM purified WT IscR, or both for 30 min at 37°C in a solution containing 40 mM Tris (pH 7.9), 30 mM KCl, 100 μg/ml BSA, and 1 mM DTT. A total of 2 μg/ml of DNase I (Worthington) was added to the reaction mix for 30 s, followed by the addition of sodium acetate and EDTA to final concentrations of 300 and 20 mM, respectively, to terminate the reaction. The reaction mix was ethanol precipitated, resuspended in urea loading dye, heated for 60 s at 90°C, and loaded onto a 7 M urea–8% polyacrylamide gel in 0.5× TBE buffer. An A+G ladder was made by formic acid modification of the radiolabeled DNA, followed by piperidine cleavage (53). The gels were visualized using a Typhoon FLA-9000 gel imaging scanner.

### Protein purification and quantification.

WT IscR was purified from strain PK8581 as previously described (22). The protein concentration was measured as described previously (24). The cluster occupancy of purified IscR was determined by precipitating the protein in an acidic solution and measuring the iron content using the 2,4,6-Tris(2-pyridyl)-s-triazine (TPTZ) method (54). Assuming that 2 mol of Fe binds per monomer of IscR, the percent occupancy of [2Fe-2S] was calculated to be between 21% and 25%.

### Molecular modeling of the IscR-RNA polymerase interaction.

The structure of IscR bound to its DNA site from P*_hyaA_* (PDB accession number 4HF1) determined by X-ray crystallography was modeled onto the cryo-electron microscopy (cryo-EM) structure of E. coli σ^70^ bound to the *rpsT* P2 promoter (PDB accession number 6PSQ) by aligning the −35 hexamer of P*_hyaA_* to that of the *rpsT* P2 promoter (24, 31), using the modeling program Sybyl (Tripos Corp.). The IscR binding site of P*_hyaA_* was transformed into the IscR binding site of P*_ydiU_* using the “mutate” function in Sybyl. The resulting model of IscR bound to its target site in P*_ydiU_* was refined using PHENIX.refine, using the PDB accession number 4HF1 electron density for the model base pairs (55). All models were energy refined using the Tripos force field through 100 cycles of minimization. The interaction between IscR and RNA polymerase at the IscR binding site of P*_ydiU_* was modeled by aligning the −35 promoter element of P*_ydiU_* to that of the *rpsT* P2 promoter.

### Statistical analysis.

Each set of *in vitro* transcription or DNase I footprinting reactions was done on three separate days, with proteins isolated on three different days. The products were visualized using a Typhoon 9000 FLA scanner, and the images were viewed using ImageJ or AzureSpot software. For *in vitro* transcription reaction quantification, the intensity of each transcript band was quantified using AzureSpot software, and automatic background correction was done using the rolling-ball method. The WT and mutant *ydiU* and *hyaA* transcript levels were normalized to the corresponding *RNA-1* levels. The normalized transcript levels from the triplicate experiments were averaged and plotted as a scatter graph. Error bars represent the standard errors of the means.

### Data availability.

All data are available within the manuscript and the supplemental material.
