# Supplementary figures and images for: Minor Alterations in Core Promoter Element Positioning Reveal Functional Plasticity of a Bacterial Transcription Factor
Source: mBio. 2021 Nov 2;12(6):e02753-21. doi: 10.1128/mBio.02753-21 (PMC8561392; doi:10.1128/mBio.02753-21)

Supplemental figure 1

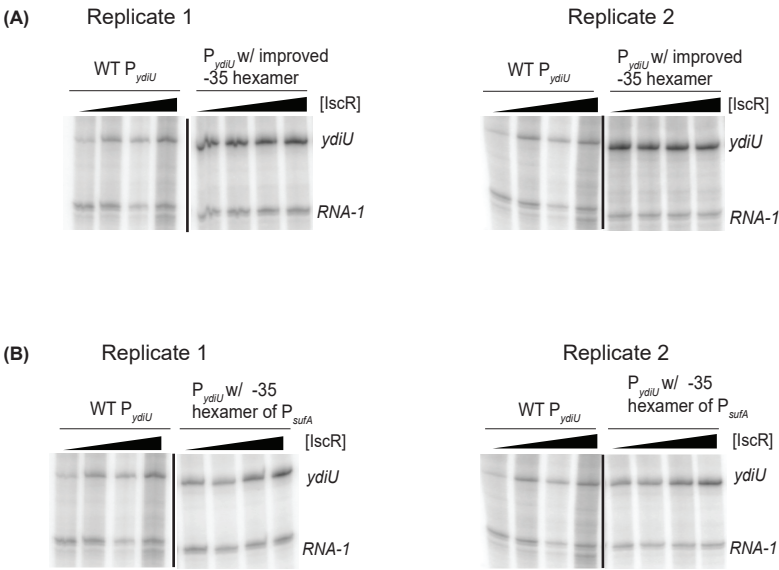

Supplement: FIG S1 [file mbio.02753-21-sf001.pdf]

Supplemental figure 2

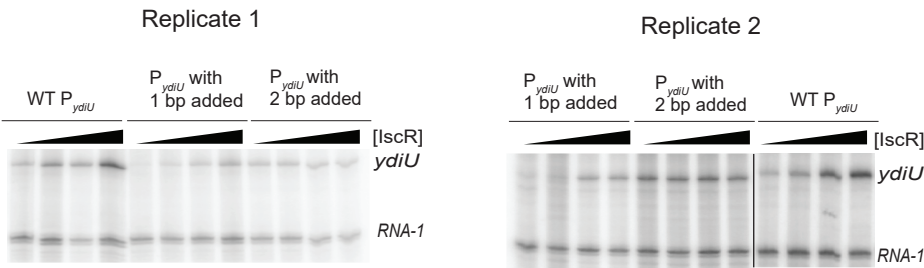

Supplement: FIG S2 [file mbio.02753-21-sf002.pdf]

Supplemental figure 3

(A)

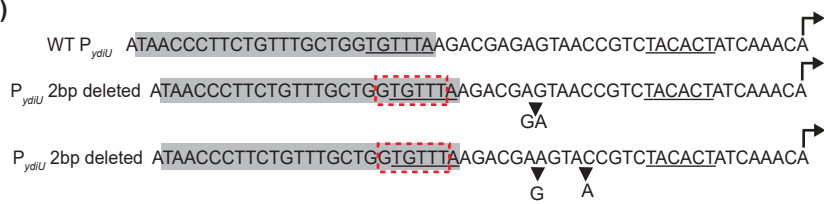

(B)

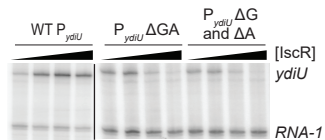

(C)

Replicate 1

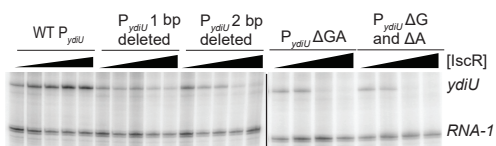

Replicate 2

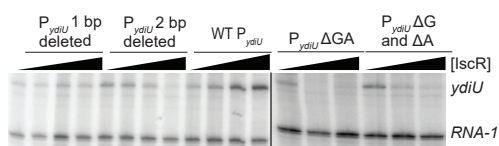

Supplement: FIG S3 [file mbio.02753-21-sf003.pdf]

Supplemental figure 4

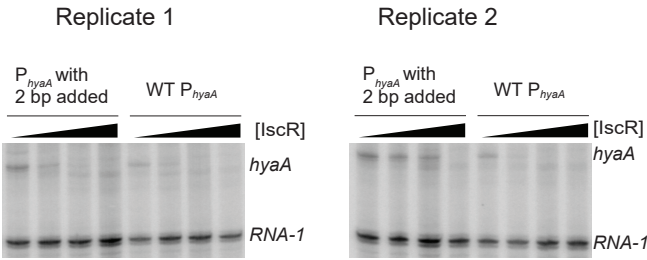

Supplement: FIG S4 [file mbio.02753-21-sf004.pdf]
