# Supplementary material for: Minor Alterations in Core Promoter Element Positioning Reveal Functional Plasticity of a Bacterial Transcription Factor
Source: mBio. 2021 Nov 2;12(6):e02753-21. doi: 10.1128/mBio.02753-21 (PMC8561392; doi:10.1128/mBio.02753-21)
Supplement: TABLE S1 [file mbio.02753-21-st001.pdf]

Supplemental Table 1: List of plasmids and *E.coli* strains used in this study

Plasmid number	Phenotype	Source
pPK7179	pUC19-spf' with XhoI site replacing SalI site	(1)
pPK13810	pPK7179 with P_{ydiU} (-200 to +40bp relative to transcription start site) cloned into BamHI and XhoI site	This study
pPK13815	pPK13810 with deletion of -17bp	This study
pPK13817	pPK13810 with deletion of -17 and -19bp	This study
pPK13818	pPK13810 with optimal -35 hexamer	This study
pPK13819	pPK13810 with -35 hexamer replaced with that of P_{sufA}	This study
pPK14063	pPK13810 with deletion of -24 and -25bp	This study
pPK14064	pPK13810 with deletion of -23 and -19bp	This study
pPK14075	pPK13810 with 1 nucleotide insertion at -17bp	This study
pPK14080	pPK13810 with 2 nucleotides insertion at -18bp	This study
pPK6842	pPK7179 with P_{hyaA} (-200 to +40bp relative to transcription start site) cloned into BamHI and XhoI site	(2)
pPK13849	pPK6842 with insertions at -17bp and -19bp	This study
pPK6161	+1 to +489 *iscR* fragment cloned into pET-11a, Ap^R	(3)
Strain number	Phenotype	Source
PK8581	BL21(DE3)*ΔhimA::tetΔiscR::kan* with pPK6161	(3)

References:

1. Kang, Y., Weber, K.D., Qiu, Y., Kiley, P.J. and Blattner, F.R. (2005) Genome-wide expression analysis indicates that FNR of Escherichia coli K-12 regulates a large number of genes of unknown function. *J Bacteriol*, **187**, 1135-1160.
2. Giel, J.L., Rodionov, D., Liu, M., Blattner, F.R. and Kiley, P.J. (2006) IscR-dependent gene expression links iron-sulphur cluster assembly to the control of O2-regulated genes in Escherichia coli. *Molecular Microbiology*, **60**, 1058-1075.
3. Fleischhacker, A.S., Stubna, A., Hsueh, K.-L., Guo, Y., Teter, S.J., Rose, J.C., Brunold, T.C., Markley, J.L., Münck, E. and Kiley, P.J. (2012) Characterization of the [2Fe-2S] cluster of Escherichia coli transcription factor IscR. *Biochemistry*, **51**, 4453-4462.
