# Supplementary material for: Minor Alterations in Core Promoter Element Positioning Reveal Functional Plasticity of a Bacterial Transcription Factor
Source: mBio. 2021 Nov 2;12(6):e02753-21. doi: 10.1128/mBio.02753-21 (PMC8561392; doi:10.1128/mBio.02753-21)
Supplement: TABLE S2 [file mbio.02753-21-st002.pdf]

Supplemental Table 2: List of primers used to construct P_{ydiU} and P_{hyaA} mutants. The XhoI and BamHI sites in the primers used for cloning WT *ydiU* into pPK7179 are underlined.

	Forward Primer, 5' to 3'	Reverse Primer, 5' to 3'
pPK13810	CCATTTCTCGAGTTCCCCTTGTTGCAA	AATTAAGGATCCCGCGCCAGCGGGTAA
pPK13818	CTTCTGTTTGCTGGTTTGAAAGACGAGAGTAACC	GGTTACTCTCGTCTTTCAAACCAGCAAACAGAAG
pPK13819	CTTCTGTTTGCTGGTTTGAAAGACGAGAGTAACC	GGTTACTCTCGTCTTTCAAACCAGCAAACAGAAG
pPK14075	GTGTTTAAGACGAGAGTAACCCGTCTACACTATCAA	TTGATAGTGTAGACGGGTTACTCTCGTCTTAAACAC
pPK14080	TTAAGACGAGAGTAAACCCGTCTACACTATCA	TGATAGTGTAGACGGGTTTACTCTCGTCTTAA
pPK13815	GCTGGTGTTTAAGACGAGAGTAACGTCTACACTATCAA	TTGATAGTGTAGACGTTACTCTCGTCTTAAACACCAGC
pPK13817	GCTGGTGTTTAAGACGAGAGTACGTCTACACTATCA	TGATAGTGTAGACGTACTCTCGTCTTAAACACCAGC
pPK13849	TTTGTATTGTTTTGTGCAAAAGGTTTTCACTACGCTTTATT	AATAAAGCGTAGTGAAAACCTTTTGCACAAAACAATACAAA
pPK14063	TGGTGTTTAAGACGAGTAACCGTCTACACT	AGTGTAGACGGTTACTCGTCTTAAACACCA
pPK14064	TGCTGGTGTTTAAGACGAAGTACCGTCTACACTAT	ATAGTGTAGACGGTACTTCGTCTTAAACACCAGCA
